# „Gesundheit fördern, heißt Demokratie fördern“: Tagungsbericht zum Kongress Armut und Gesundheit 2025

**DOI:** 10.1007/s00103-025-04128-z

**Published:** 2025-09-11

**Authors:** Nicole Böhme, Dominica Czora, Julia Demirdizen, Maren Janella, Marina Martin, Jens Hoebel

**Affiliations:** 1 Geschäftsstelle Berlin, Gesundheit Berlin-Brandenburg e. V., Berlin, Deutschland; 2 Fachgebiet Soziale Determinanten der Gesundheit, Robert Koch-Institut, Nordufer 20, 13353 Berlin, Deutschland

Am 17. und 18.03.2025 fand der 30. Kongress Armut und Gesundheit statt – erneut im Henry-Ford-Bau an der Freien Universität Berlin. Dass Armutslagen auch in einem wohlhabenden Land wie Deutschland mit schlechteren Gesundheitschancen verknüpft sind, ist u. a. durch die Gesundheitsberichterstattung umfassend beschrieben. Laut einer aktuellen Studie des Robert Koch-Instituts liegt zum Beispiel die Lebenserwartung in den sozioökonomisch am stärksten benachteiligten Regionen 4,3 Jahre (Frauen) beziehungsweise 7,2 Jahre (Männer) niedriger als in den wohlhabendsten Regionen Deutschlands. Die Daten zeigen, dass sich diese gesundheitliche Ungleichheit über die letzten Jahrzehnte vergrößert hat [1]. Beim Kongress wurde in über 90 Veranstaltungen und mit knapp 1400 Teilnehmenden diskutiert, wie gesundheitliche Ungleichheiten verringert und eine gerechtere, gesündere Gesellschaft geschaffen werden können – auch unter Berücksichtigung der aktuellen Klima- und Umweltveränderungen. In diesem Tagungsbericht fokussieren wir auf die Veranstaltungen, die explizit Bezug zum Themenschwerpunkt „Demokratieförderung“ genommen haben.

Gesundheitsförderung ist ein Demokratieprojekt! Es gibt wechselseitige Beziehungen zwischen demokratischen Prozessen und Gesundheitsförderung: Die Stärkung demokratischer Prozesse fördert das gesellschaftliche Bewusstsein für gesundheitliche Chancengleichheit, die Selbstbestimmung und Mitbestimmung. Durch die aktive Teilnahme an gesellschaftlichen Prozessen erfahren Menschen Selbstwirksamkeit, stärken ihr Vertrauen in Institutionen und erleben soziale Unterstützung. All diese Faktoren sind wesentliche Determinanten für die Gesundheit. Umgekehrt kann Gesundheitsförderung dazu beitragen, die gesellschaftliche Teilhabe zu erhöhen, indem sie Menschen befähigt, ihre Rechte einzufordern und sich aktiv am gesellschaftlichen Leben zu beteiligen. Der Kongress Armut und Gesundheit 2025 tagte daher unter dem Motto: „Gesundheit fördern, heißt Demokratie fördern“.

## Unser Gesundheitsförderungsverständnis braucht ein neues Demokratieverständnis

Durch Wahlen bestimmen Bürger*innen in Deutschland ihre politischen Vertreter*innen, die im Parlament die Verantwortung übernehmen, die Rahmenbedingungen des Gemeinwesens zu gestalten und die Daseinsvorsorge sicherzustellen (vgl. hier und im Folgenden Diskussionspapier zum Kongress Armut und Gesundheit 2025 [2]). Wenn Bürger*innen das Gefühl haben, Politiker*innen kommen dieser Verantwortung nicht nach, untergräbt dies die staatliche Legitimation und das Vertrauen in staatliche Einrichtungen. Sozial ungleiche Umwelt- und Lebensbedingungen sowie damit verbundene Ungleichheiten in den Gesundheitschancen können den Eindruck verstärken, dass der Staat in seiner derzeitigen Verfassung nicht stark genug ist, um aktuellen und künftigen Herausforderungen zu begegnen. Ein funktionsfähiger Staat, der seinen Bürger*innen u. a. gesundheitliche Chancengleichheit ermöglicht (Daseinsvorsorge, Diskriminierungsverbot) und alle Betroffenen gleichermaßen in politische Entscheidungen einbezieht, kann zu mehr Demokratievertrauen beitragen [2].

Um das soziale Miteinander, den gesellschaftlichen Zusammenhalt und die individuelle wie auch kollektive Gesundheit zu stärken, ist die Förderung von Partizipation und Mitbestimmung essenziell. Demokratieförderung und die Bekämpfung von Armut sind eng miteinander verknüpft und es ist unsere gemeinsame Aufgabe, diese Wechselbeziehung in den Mittelpunkt unserer Bemühungen zu stellen.

Den diesjährigen Kongress eröffnete Maren Urner, Professorin für Nachhaltige Transformation (FH Münster). Sie leitete ein mit der Frage: „Was müsste passieren, damit ich mein Leben verändere?“ Sie machte deutlich, dass gesellschaftliche und politische Veränderungen nicht allein rational erklärbar sind, sondern immer auch eine emotionale Dimension besitzen. Daraus leitete sie eine „Reifeprüfung“ für das Individuum ab, die auf 3 Ebenen angesiedelt ist:

1. *Emotionale Reife* durch radikale Aufmerksamkeit. Urner sprach von einer verzerrten Wahrnehmung. Unser Gehirn sei dazu gemacht, zu überleben, nicht um die Realität gut abzubilden; negative Nachrichten würden daher höher bewertet. Wir müssten lernen, Emotionen wahrzunehmen und diese anzuerkennen, auch die negativen. Die Kernfrage für die Veränderung sollte lauten: Wofür statt wogegen sind wir?
2. *Kommunikative Reife* durch radikale Ehrlichkeit – um verzerrte Narrative zu überwinden. Wir würden uns die falschen Geschichten darüber erzählen, „was normal ist“. „Warum zerstören wir unsere Lebensgrundlage, obwohl wir es eigentlich besser wissen?“ Der Grund hierfür seien Machtungleichgewichte auch im Hinblick auf Wissen. Wir müssen realitätsnähere Geschichten erzählen. Eine dieser realistischen Geschichten ist der Mensch als soziales Wesen, dessen Stärke in Kooperation liegt statt in Konkurrenz.
3. *Soziale Reife* durch radikale Verbundenheit zur Überwindung von gesellschaftlichen „Trennlinien“ (emotional vs. rational, privat vs. politisch, Wirtschaft vs. Klimaschutz) und als Voraussetzung für kollektives Handeln.

Susanne Hartung, Professorin an der Hochschule Neubrandenburg, betonte: „Viele Menschen fühlen sich nicht mehr wirklich gehört. Demokratie steht vor großen Herausforderungen und bei vielen Menschen entsteht ein Gefühl der Entfremdung, auch gegenüber politischen Institutionen“, so Hartung weiter. Mit Zuversicht blickte sie nach vorn, da partizipative Ansätze zunehmend an Bedeutung gewinnen und Handlungsansätze bieten. Die Leitfrage der von Wissenschaft, Praxis und Menschen mit Armutserfahrung gemeinsam entwickelten Veranstaltung lautete daher: „Demokratie stärkt Partizipation – stärkt Partizipation Demokratie?“

Im Dialog mit Menschen mit Armutserfahrungen wurde zunächst diskutiert, worin die Motivation besteht, sich an partizipativen Prozessen zu beteiligen – für Menschen in Armutslagen, aus der Praxis und der Wissenschaft. Wissenschaftler*innen nannten als Motivation den Anspruch, tatsächlich Veränderungen zu bewirken – und das sei nur im Dialog mit Betroffenen möglich. Menschen in Armutslagen ginge es vor allem um Teilhabe. Als gutes Praxisbeispiel wurde die 1991 als deutsche Sektion des Europäischen Armutsnetzwerks EAPN (European Anti Poverty Network) gegründete „Nationale Armutskonferenz“ (nak) benannt. Jürgen Schneider, engagiert im Koordinationskreis der Nationalen Armutskonferenz, betonte, wie wichtig ein ehrliches Interesse potenzieller Unterstützer*innen ist. Partizipation kann wirksam sein, wenn sie wirklich ernst gemeint ist und Menschen in Armutslagen wertfrei gehört und ernst genommen werden. Erika Biehn (Verband alleinerziehender Mütter und Väter Landesverband Bremen e. V.) ergänzte: „Nur weil ich arm bin, heißt das nicht, dass ich nichts mehr kann.“ Aus Sicht der kommunalen Praxis wurde auch betont, wie wertvoll der Aufbau von Wissen um partizipative Methoden und die Langfristigkeit von Strukturen der kontinuierlichen Zusammenarbeit zwischen Wissenschaft, Praxis und Betroffenen seien.

## Partizipation als gelebte Demokratie

Mehrere weitere Veranstaltungen widmeten sich dem Verhältnis von Partizipation und Demokratie. In der Veranstaltung „Gelebte Demokratie – wer ist Teil demokratischer Prozesse? Chancengerechte und partizipative Angebote gestalten“ wurde herausgestellt, dass Partizipation zur Erweiterung und Vertiefung der Demokratie beitragen kann, da sie die Prinzipien demokratischer Gesellschaftsgestaltung auf zahlreiche Lebenswelten ausweitet (Izabela Bojkowska, Hessische Arbeitsgemeinschaft für Gesundheitsförderung e. V.). Als Beispiel, wie Gesundheitsförderungsangebote zur Förderung demokratischer Teilhabe beitragen können, nannte Izabela Bojkowska Stadtteilspaziergänge. Dabei untersuchen Teilnehmende den Stadtteil, z. B. mit „Photovoice“, einer Methode, bei der Eindrücke durch Fotografien dokumentiert und gemeinsam reflektiert werden. Die Ergebnisse können anschließend im Rahmen eines demokratischen Prozesses in die Entscheidungen des Grünflächen- oder Städtebauamtes einfließen. Gerade in Gesundheitsförderungsprojekten können so insbesondere Menschen, die häufig aufgrund von Benachteiligung eher von demokratischer Teilhabe ausgeschlossen seien, in diese Prozesse einbezogen und damit auch deren Zustimmung zu demokratischen Prinzipien gestärkt werden. Linda Huf-Hoko (Hessische Arbeitsgemeinschaft für Gesundheitsförderung e. V.) führte aus, dass Gesundheitsförderungsangebote durch ihre Rückkopplung an die Lebenswelt der Menschen ein zentrales Element sozialer Gerechtigkeit und demokratischer Teilhabe seien.

Beteiligungsverfahren wurden auch im Rahmen der Veranstaltung: „Partizipation und Demokratie – Erfahrungen aus dem Projekt Kontextcheck“ hinsichtlich ihrer Auswirkungen auf die Demokratie diskutiert. Das Projekt Kontextcheck begleitet Kommunen in Niedersachsen dabei, Rahmenbedingungen für Gesundheitsförderung vor Ort systematisch zu erfassen und integrierte kommunale Strategien zu formulieren. Dabei sind Verfahren zur Beteiligung von Bürger*innen ein wichtiges Mittel. Selbstkritisch wurde hinterfragt, wie das Verhältnis von Partizipation im Rahmen solcher Beteiligungsverfahren zur repräsentativen Demokratie sei und ob diese vielleicht sogar negative Auswirkungen im Sinne u. a. von Vertrauensverlusten haben können, wenn aus den Beteiligungsverfahren nichts resultiere.

### Gesellschaftliche Teilhabe für Armutsbetroffene

Im Rahmen der Veranstaltung des Gremiums der Menschen mit Armutserfahrungen, das den Kongress begleitete, wurde betont, dass gesellschaftliche Teilhabe weit über die Stimmabgabe bei Wahlen hinausgeht. Demokratische Mitwirkung setzt Ressourcen voraus – Zeit, Geld, Zugang zu Räumen, emotionale Sicherheit. Menschen in prekären Lebensverhältnissen können sich die Teilnahme an Veranstaltungen jedoch oft nicht leisten. Schon der Besuch einer öffentlichen Sitzung scheitert mitunter an der unausgesprochenen Erwartung, im Veranstaltungsraum Speisen oder Getränke zu erwerben. Das dafür im Regelbedarf vorgesehene Budget ist gering und vielfach bereits durch andere notwendige Ausgaben aufgebraucht. Diese symbolisch „kleine“ Hürde stehe beispielhaft für strukturelle Ausschlüsse, die selten benannt werden.

Die Journalistin und Autorin Mareice Kaiser griff diese Perspektive auf. In Redaktionen habe sie erlebt, dass solche Themen abgelehnt würden, weil sie als „nicht relevant“ erschienen – was in der Praxis häufig bedeute: nicht im Erfahrungshorizont der Entscheidenden. „Menschen mit Geld kaufen Bücher und entscheiden über Inhalte. Wer Armut nicht kennt, interessiert sich nicht für ihre Geschichten.“ Zudem wies Kaiser auf die strukturelle Gewalt hin, der armutsbetroffene Menschen ausgesetzt sind, wenn sie öffentlich über ihre Lage sprechen. Sie verwies dabei auf den in sozialen Medien verbreiteten Hashtag #IchBinArmutsbetroffen, unter dem massiver Hass und Drohungen gegen Betroffene sichtbar wurden. Ihre Schlussfolgerung: „Unsere Gesellschaft verachtet Armut – und braucht dringend andere Narrative.“

Professor Christoph Gille (Hochschule Düsseldorf) hat Beteiligungsformate armutsbetroffener Menschen im europäischen Vergleich untersucht. Belgien etwa verpflichtet sich gesetzlich zur Beteiligung von Menschen mit Armutserfahrung. In Portugal existieren 18 regionale runde Tische mit direktem Politikzugang. Deutschland hingegen verfüge – trotz jahrzehntelanger Arbeit von Gremien wie der Nationalen Armutskonferenz – kaum über gesicherte Strukturen. Selbstvertretung sei wichtig, aber ohne Ressourcen bleibe sie „zahnlos“. Auch die Überwindung von Scham und die Bedeutung gemeinsamer politischer Teilhabe kamen zur Sprache. Jürgen Schneider warnte in diesem Zusammenhang vor der derzeitigen Verwendung des Begriffs „Selbstvertretung“. Er wies darauf hin, dass viele Menschen sich diesen Begriff aneigneten, ohne eigene Betroffenheit oder Erfahrung einzubringen. Der Begriff „Selbstorganisation“ sei in diesem Kontext treffender und wirke einer Entwertung entgegen. Mareice Kaiser formulierte: „Wir könnten Armut abschaffen. Es gibt genug Geld, nur ist es ungleich verteilt. Statt über Moral müsse über Macht gesprochen werden und über Reichtum.“ Christoph Gille ergänzte, dass wir das „meritokratische Prinzip“ grundsätzlich hinterfragen müssten – die verbreitete Vorstellung, dass jede*r genau das bekommt, was er oder sie verdient. Diese Grunderzählung des Kapitalismus legitimiere bestehende Ungleichheit und ermögliche sozialen Ausschluss, da Armut mit Leistungsschwäche gleichgesetzt würde. Demokratie dürfe nicht auf solchen Leistungsideologien gründen. „Wir alle sind verletzlich. Und daraus erwächst ein Recht auf Teilhabe.“

### Demokratie in Bezug auf die Gesundheit von Kindern und Jugendlichen

Der Zusammenhang zwischen Gesundheit und Demokratie wurde in verschiedenen Veranstaltungen auch über bestimmte Lebensphasen hinweg thematisiert, beispielsweise in der Auftaktveranstaltung der Frühen Hilfen. Professor Jörg Fischer (erweiterter Vorstand des Beirats der Bundesstiftung Frühe Hilfen und des Nationalen Zentrums Frühe Hilfen) bezeichnete Frühe Hilfen als einen „Start in ein demokratisches Miteinander“. Zugleich räumte er ein, dass dies keine selbstverständliche Auslegung sei. So existierten etwa auch „völkische Ideen“ zu den Frühen Hilfen, die es erforderlich machten, eigene implizite Annahmen offenzulegen, Position zu beziehen und die Diskussion zu suchen, so Fischer weiter: „Die Frühen Hilfen brauchen ein stärkeres Selbstverständnis, um sich zielgerichteter nach außen darzustellen, sich proaktiv als Teil einer demokratischen Kultur zu begreifen. Und sie brauchen auch eine Wertschätzung, die ihnen entgegengebracht und von ihnen weitergetragen wird, gegenüber den Menschen, gerade vor dem Hintergrund, dass sich die Frühen Hilfen nicht ideologisch vereinnahmen lassen sollen. … Die Frühen Hilfen sind aber auch Teil einer bürgerlichen Pflicht, unseren Staat, unsere Form des Zusammenlebens zu schützen, manchmal auch sogar zu verteidigen, damit wir weiterhin für alle da sein können.“

Professorin Ute Thyen (Vorsitzende des Beirats der Bundesstiftung Frühe Hilfen und des NZFH) führte an, dass zur Stärkung von Familien auch die Stärkung der Kinderrechte gehöre. Dazu zähle beispielsweise auch ein Wahlrecht für Kinder. Bei Kindern, die noch nicht in der Lage seien oder sich nicht in der Lage fühlten, diese Entscheidung selbst zu treffen, könne die Wahl auch stellvertretend durch die Erziehungsberechtigten im besten Sinne des Kindes erfolgen. Es sei auch davon auszugehen, dass Parteien daraufhin ihre Wahlprogramme anpassen und die Belange von Kindern stärker berücksichtigen würden.

In den verschiedenen Veranstaltungen wurde der Aspekt der Demokratie in Bezug auf die Gesundheit bei Kindern und Jugendlichen aus ganz unterschiedlichen Perspektiven diskutiert. Beispielsweise berichteten Julia Brüggemann und Maja Kuchler von der Hochschule Bochum von Kinder- und Jugendkonferenzen in Berlin, Eisenach und Chemnitz, die 2023 und 2024 im Rahmen des von der Bertelsmann Stiftung geförderten Projektes „Politik vom Kind aus denken“ durchgeführt wurden, um die Partizipation von Kindern und Jugendlichen an politischen Prozessen zu stärken und ihre Bedarfe, Wünsche und Forderungen für ein gesundes Leben zu erheben. In angeleiteten Workshops, in denen zunächst ein gemeinsames Grundverständnis von Gesundheit erarbeitet wurde, formulierten die teilnehmenden Kinder und Jugendlichen in Untergruppen ihre gesundheitsbezogenen Forderungen. So fand neben der inhaltlichen Auseinandersetzung mit Gesundheit auch das Erleben des Prozesses von Aushandlungen untereinander und Entscheidungsfindung statt. Zu den formulierten Forderungen zählten unter anderem die Weiterbildung von Lehrkräften und sozialen Diensten zu Themen wie Diskriminierung sowie strukturelle Veränderungen zur Verstärkung des sozialen Zusammenhalts. Ebenso müssten medizinische Versorgung und Prävention verbessert werden. Die Teilnahme am Kinder- und Jugendrat trug zum Empowerment der Beteiligten und zur Demokratieförderung bei – „allerdings nur dann, wenn kommunale Politiker*innen da sind und das weitertragen“ (Julia Brüggemann).

Einen anderen Ansatz wählt Pater Oliver Potschien (Abtei Hamborn). Er berichtete von seinen Erfahrungen mit „libanesischen Clan-Jugendlichen“ in Duisburg-Marxloh. Seit 2015 werde ein falsches und vorurteilsbehaftetes Bild von Jugendlichen mit Migrationshintergrund gezeichnet, welches empirisch nicht haltbar sei, aber dennoch negative Auswirkungen auf die Jugendlichen habe. Seine Erfahrung zeigte, dass ein machtkritischer Ansatz – also die bewusste Auseinandersetzung mit Vorurteilen, Diskriminierung und ungleichen Machtverhältnissen – und gezielte Angebote für die Jugendlichen sowohl gesundheitliche Potenziale entfalten als auch zur Entspannung der konfliktbelasteten Situation im Duisburger Norden beitragen konnten. In der Folge engagieren sich Jugendliche, in deren Umfeld es in der Silvesternacht 2022/2023 zu Übergriffen gegen Einsatzkräfte gekommen war, selbst als ausgebildete Rettungshelfer und „Stadtteilsanitäter“ in Marxloh.

Eine ähnlich machtkritische Perspektive verfolgt auch das Projekt „JuPoint Treffpunkte“, welches von Per Traasdahl (Cajiu e. V.) vorgestellt wurde. Er berichtete von einem „Umbau alltagstrivialer Machtfaktoren“ wie Schlüsselgewalt oder Öffnungszeiten, durch den Partizipation und Begegnungen auf Augenhöhe ermöglicht werden. Bestimmte Gruppen von Jugendlichen zeigten eine geringe Akzeptanz von Angeboten. Kommunikation beginne nicht, wie oft angenommen, erst bei der persönlichen Begegnung, sondern bereits bei den materiellen Gegebenheiten vor Ort. „Da kommen so viele Signale, so viele Vorbestimmungen mit rein und da steht auch Macht dahinter. Deshalb haben wir überlegt: Kann man auch anders anfangen?“ (Per Traasdahl). So wurde ein alter Bienenwagen auf einer Wiese im Park zunächst gemeinsam mit Förderschüler*innen umgebaut und dann zum Mittelpunkt des Projektes. Schließlich akzeptierten die Jugendlichen das Angebot, trotz bürokratischer Hürden und Voraussetzungen die einer Anerkennung als Jugendfreizeiteinrichtung eigentlich entgegenstünden.

Volker Kersting (Verein für Sozialplanung, VSOP e. V.) brachte eine neue Perspektive ein, indem er die Rolle der kommunalen Verwaltung hervorhob. Ausgangspunkt seiner Ausführungen waren nachweisbare Zusammenhänge zwischen Armut, Gesundheit und demokratischer Teilhabe, die sich unter anderem an der Wahlbeteiligung zeigen. Solche Zusammenhänge lassen sich mithilfe kommunaler Routinedaten (Schuleingangsuntersuchungen, Daten zur Grundsicherung und Wahlbeteiligung) auch kleinräumig darstellen. Für Mülheim an der Ruhr führte er aus, dass dort die Schuleingangsuntersuchungen um einen Fragebogen ergänzt wurden, der soziale Kontexte des Aufwachsens wie Kita-Zeiten, Freizeitverhalten, Mitgliedschaft im Sportverein oder musikalische Früherziehung abfragte. Er appellierte dabei an die Teilnehmenden: „Ich empfehle Ihnen allen, das in Ihre Kommunen zu transportieren, da es ein hervorragendes und wichtiges Instrument ist.“ Die abgefragten Faktoren wurden mit den Kompetenzen der Kinder in Beziehung gesetzt und statistisch analysiert, wie diese sich auf die Kinder auswirken. Anschließend wurde herausgearbeitet, auf welche der Faktoren Einfluss durch die Kommune genommen werden kann, um kommunale Hebel für die Förderung der Kinder zu identifizieren. Förderfaktoren, auf die die Kommunen Einfluss haben, sind beispielsweise der Zugang zur Kita-Betreuung und zu Sportvereinen oder die Förderung eines Familienzentrums. „Die erwarteten Effekte einzelner Faktoren wurden modellhaft berechnet und überall da, wo wir uns mit einer Förderung durchsetzen konnten, haben wir massive Effekte erlebt“, so Kersting. Kommunale Praktiken müssten also vor Ort anhand solcher Analysen ausgerichtet werden. Ein konkretes Beispiel hierzu können kommunale Sportgutscheine sein.

### Demokratie und ältere Menschen

Doch auch im Alter sind Demokratie und Gesundheit eng miteinander verbunden. Sozial- und Wohlfahrtsstaatlichkeit als zentrale Grundprinzipien einer Demokratie wurden gemeinsam in der gleichnamigen Veranstaltung über „Verfügbarkeit und Passung von Leistungen im Pflegefall“ diskutiert. Anhand von Fallanalysen wurden komplexe Bedarfe der Betroffenen sowie Lücken und Brüche in der Versorgung thematisiert.

Melina Maier (Institut für Gerontologische Forschung e. V.) stellte hierzu beispielhaft 3 inhaltsanalytisch aufgearbeitete Fälle vor. Der Blick auf einzelne Fälle sei ein wichtiger Zugang zur Versorgungsrealität. Professorin Anja Dieterich und Professorin Julia Franz (beide Alice Salomon Hochschule Berlin) führten aus, dass sich Probleme und Potenziale so systematisch erfassen und als strukturelle Missstände in die Politik einbringen lassen, um Reformen anzustoßen – denn trotz eines theoretischen Anspruchs sei die Versorgung im Pflegefall in der Praxis noch immer nicht ausreichend gewährleistet.

Barbara Susec (ver.di-Bundesverwaltung), die auch die Perspektive der Beschäftigten einbrachte, resümierte, dass aus dem Eindruck, der Staat komme seiner Aufgabe, die Pflege zu gestalten, nicht nach, auch Demokratieverdrossenheit resultieren könne. Sie schloss mit der Hoffnung, die neue Bundesregierung gehe eine umfassende Pflegereform an und führte deren Eckpunkte aus: „Wir brauchen einen Wettbewerb um die Qualität statt um den Preis – und dafür eine grundlegende Reform der Finanzierung. Unser Konzept dafür nennen wir ‚solidarische Pflegegarantie‘. Alle zahlen entsprechend ihres Einkommens ein, also auch Erträge aus Kapitaleinkommen müssen einbezogen werden. Wir können uns das Nebeneinander von privater und sozialer Pflegekasse nicht mehr leisten.“

### Diskriminierung entgegenwirken

In der Veranstaltung: „Diskriminierung in der Gesundheitsversorgung strukturell entgegenwirken“, standen unterschiedliche Perspektiven im Mittelpunkt: Tuğba Yalçınkaya (Charité – Universitätsmedizin Berlin) beleuchtete die Auswirkungen von Diskriminierung in der Gesundheitsversorgung, Anthea Backfisch und Sybill Schulz, ebenfalls Charité – Universitätsmedizin Berlin, stellten Handlungsansätze für diversitätsgerechte Strukturen in Kliniken vor. Dr.in Ute Siebert (Charité – Universitätsmedizin Berlin) zeigte Möglichkeiten zum Abbau von Diskriminierung durch Qualifizierung auf und Ksenia Meshkova (Hochschule Fulda) präsentierte Bedingungen für organisationale Interventionen gegen Rassismus in Einrichtungen der Gesundheitsversorgung.

Zahlreiche Studien belegen, dass Menschen, die Diskriminierungen im Alltag erleben oder rassifiziert werden, in der Gesundheitsversorgung benachteiligt sind. Sie erhalten häufiger eine schlechtere Behandlung z. B. durch Fehlversorgung oder Ausschlussmechanismen, wobei Sprachbarrieren am häufigsten genannt werden. Dies führt zu einem Vertrauensverlust gegenüber dem Gesundheitssystem und löst eine Abwärtsspirale aus, die die Gesundheit dieser Personengruppe nachhaltig beeinträchtigt (vgl. [3]). Diese intersektionalen Diskriminierungsformen wurden von den Beitragenden an konkreten Handlungsbeispielen aus der Praxis unter der Frage: „Wie können wir Diskriminierung in der Gesundheitsversorgung gezielt entgegenwirken?“, diskutiert.

### Austausch zwischen Politik, Wissenschaft und Praxis

„Gesundheitsförderung braucht Evaluationen. Gesundheitsförderung braucht Qualitätsentwicklung und -sicherung. Gesundheitsförderung braucht Nachweise ihrer Wirkungswirksamkeit.“ Mit diesen 3 Forderungen ging es ins Gespräch zur Frage, welche Bedingungen erfüllt sein müssen, um den Austausch zwischen Politik, Wissenschaft und Praxis voranzutreiben. Zunächst wurden Barrieren zur Umsetzung von evidenzbasierten Beratungen identifiziert, u. a. fehlende Kompetenzen im Öffentlichen Gesundheitsdienst (ÖGD), um Bedarfe und deren Anwendung identifizieren zu können. Aber auch knappe zeitliche, finanzielle oder personelle Ressourcen, fehlende Drittmittel für Qualitätssicherung und Evaluation von Maßnahmen seien hinderlich. Dies verlange vor allem eine Offenlegung von Entscheidungsgrundlagen in der Planung und Umsetzung der Vorhaben. Transparenz wurde von Marion Chenevas (Gesundheitsreferat, Landeshauptstadt München) hierfür als grundlegend benannt.

Weiterhin reflektierte Eva Rehfuess (Ludwig-Maximilians-Universität München), dass „guter Austausch zwischen Wissenschaft und Praxis sowie gemeinsame Forschung nur dann gelingen, wenn man von Anfang an im gleichen Boot sitzt.“ Damit plädierte sie nicht nur für einen transparenten Wissenschafts-Politik-Transfer, sondern auch für langfristige partizipative Forschung. Diese ermögliche es, Gesundheitsdaten über einen langen Zeitraum zu bewerten und Maßnahmen flexibel an veränderte Bedarfe anzupassen.

Abschließend wurde gefordert, Gesundheitsförderung nicht nur als eine Säule im Gesundheitswesen zu verankern, sondern als Dach oder Fundament, das Gesundheitssicherung und Abbau sozialer Ungleichheit gleichermaßen zum Ziel hat.

### Eine sozial gerechte ökologische Transformation

Die Abschlussveranstaltung des Kongresses unterstrich, dass Public Health, Umweltinstitutionen, soziale Bewegungen und Medien gemeinsam für eine sozial gerechte ökologische Transformation eintreten müssten. Ziel sei es, positive Zukunftsbilder zu entwickeln und komplexe Zusammenhänge verständlicher zu kommunizieren.

Professor Dirk Messner (Umweltbundesamt) machte deutlich, dass die sozialökologische Transformation nicht nur technologische, sondern vor allem soziale und kommunikative Herausforderungen mit sich bringt. Umweltschutz und Gesundheit müssen zusammen gedacht werden, da die Zerstörung von Ökosystemen direkte Auswirkungen auf die menschliche Gesundheit hat. Trotz der weiterhin breiten Unterstützung für nachhaltige Veränderungen befürchten viele Menschen soziale und wirtschaftliche Nachteile. Laut Messner sei es möglich, die Klimaziele bis 2030 zu erreichen. Er plädierte für politische Klarheit, soziale Maßnahmen und eine verständliche Kommunikation. Lisa Groß von Fridays for Future beschrieb die derzeitige schwierige Resonanz auf klimapolitisches Engagement und den Einfluss populistischer Narrative, die Klimaschutz abwerten. Die Zustimmung zum Klimaschutz sei zwar weiterhin vorhanden, aber man müsse wieder einfacher, nahbarer und klarer über die Zusammenhänge sprechen. Ihr Appell: „Demokratie lebt vom Mitmachen – und Klimaschutz geht nur in einer stabilen, solidarischen Demokratie.“

### Positive Kommunikationskultur

Stefanie Bovermann (Nachwuchsnetzwerk Öffentliche Gesundheit) unterstrich die Rolle des Öffentlichen Gesundheitsdienstes (ÖGD) in der Vertrauensbildung vor Ort. Gerade kommunale Strukturen seien ein Schlüssel, um Demokratie erfahrbar und die Auswirkungen politischer Maßnahmen verständlich zu machen. Sie sprach sich für eine positivere Kommunikationskultur in der Public-Health-Community aus, die nicht nur Defizite, sondern auch Chancen und Lösungen in den Mittelpunkt rücke. Dafür brauche es eine stärkere transdisziplinäre Zusammenarbeit, über Sektoren und Verwaltungsebenen hinweg.

Jürgen Schneider wies auf die Verantwortung der Medien bezüglich der Darstellung von Menschen mit Armutserfahrungen hin. Menschen würden oft nur dann gehört, wenn sie ihre Not „ausstellen“, nicht aber, wenn sie politische Ideen einbrächten. Der Begriff der Selbstorganisation sei für ihn hierbei zentral, im Sinne gesellschaftlicher Teilhabe. Okan Bellikli (Table.Briefings) betonte die Verantwortung der Medien in emotional geführten Sozialpolitik-Debatten, die von Teilen der Politik instrumentalisiert würden, faktenbasiert zu berichten. Wichtig sei auch, auf die Sprache zu achten – indem man zum Beispiel Formulierungen wie „sozial schwach“ vermeidet.

## Ausblick auf den Kongress Armut und Gesundheit 2026

Schon heute laden wir Sie herzlich ein, sich den Termin für den kommenden Kongress vorzumerken: – den 16. und 17.03.2026 im Henry-Ford-Bau der Freien Universität in Berlin-Dahlem. Den Zusammenhang zwischen Demokratie und Gesundheitsförderung behalten wir weiterhin im Blick, ebenso wie das Thema Health in All-Policies. In 2026 tagen wir unter dem Motto: „Gesundheit ist politisch! Was ist uns Chancengerechtigkeit (als Gesellschaft) wert?“ Ab Dezember 2025 können Sie sich unter www.armut-und-gesundheit.de anmelden.

### Infobox Kongress Armut und Gesundheit

Website: www.armut-und-gesundheit.de

Bluesky: https://bsky.app/profile/kongress-aug.bsky.social

Mastodon: https://berlin.social/deck/@kongress_armut_und_gesundheit

LinkedIn (Gesundheit Berlin-Brandenburg e. V.): https://www.linkedin.com/company/gesbb
